# Sources of dietary recommendations and adherence to clinical guidelines in pregnant women in Germany

**DOI:** 10.1186/s12884-025-08228-1

**Published:** 2025-10-13

**Authors:** Alexandra Hett, Martin Smollich

**Affiliations:** Institute of Nutritional Medicine, University Hospital Schleswig-Holstein, Luebeck, Germany

**Keywords:** Pregnancy, Nutritional recommendations, Nutrition guidelines, Fetal health, Maternal nutrition, Dietary counseling, Nutrition guideline adherence, Health information sources, Social media, Prenatal care

## Abstract

**Background:**

Nutrition during pregnancy affects the health of both the mother and the child with potential long-term consequences. Consequently, educating pregnant women about recommended nutritional practices is essential. This cross-sectional study examined dietary information sources used by pregnant women in Germany and evaluated their adherence to clinical guidelines.

**Methods:**

Pregnant women (≥ 6th week of gestation) were recruited nationwide via social media (January–June 2022), and completed an online questionnaire assessing sources and content of their nutritional information. The data were subsequently analyzed for their alignment with guidelines provided by the Germany-wide Healthy Start Network of the Federal Center for Nutrition and the German Nutrition Society. Chi-square and t-tests were used to explore associations, and multivariable logistic regression was conducted to identify significant predictors of supplement use and dietary practices, considering sociodemographic and behavioral factors.

**Results:**

Of 3,363 participants, 68.66% reported dietary changes during pregnancy, yet 82.76% received limited or no counseling from healthcare providers. Most women (77.70%) sought information online. While 89.03% took dietary supplements, this percentage was significantly higher among pregnant women with higher income (OR = 1.55, CI 1.18–2.03, *p* < 0.001) and higher educational levels (OR = 1.47, CI 1.15–1.88, *p* = 0.001). Although most of the women (87.87%) supplemented with folic acid, only 66.10% supplemented with iodine. However, when considering dosage and supplementation period, at least 37.08% of women for folic acid and 46.06% for iodine did not meet recommendations or did not supplement at all.

**Conclusions:**

While recommendations to avoid specific foods and to take a folic acid supplement are widely recognized, the dietary practices of pregnant women frequently deviate from professional guidelines. These results emphasize the importance of targeted education and standardized counseling by qualified professionals to ensure optimal nutrition during pregnancy and reduce potential risks to both mother and child. Future research should identify strategies to effectively address these problems.

**Supplementary Information:**

The online version contains supplementary material available at 10.1186/s12884-025-08228-1.

## Background

Pregnancy is a crucial period for the health of both the mother and the child. Adequate nutrition is essential for ensuring the development and well-being of both individuals, with potential long-term health consequences, including a decreased risk of metabolic disorders such as diabetes and cardiovascular diseases [1–3]. The German Nutrition Society (hereinafter “the DGE”) and the Germany-wide Healthy Start Network of the Federal Center for Nutrition (BZfE) provide guidelines for nutrition during pregnancy [4, 5]. These recommendations aim to ensure that the expectant mother maintains a balanced and healthy diet, thereby ensuring that the unborn child receives all essential nutrients.

During pregnancy, increased energy and nutrient intake support maternal and fetal needs [6, 7]. The DGE suggests no increased energy intake in the first trimester, 250 kcal in the second, and 500 kcal in the third [8]. Yet, many pregnant women in industrialized nations exceed the recommended weight gain due to higher caloric intake and less physical activity, which increases their risk of gestational diabetes mellitus (GDM) and preeclampsia [9–12]. For this reason, the German Nutrition Society (DGE) recommends incorporating physical activity into daily routines, aiming for at least 30 min five times per week, with more being encouraged. In addition to energy intake, the adequate intake of micro- and macronutrients is vital, especially iron, folic acid, iodine, omega-3 fatty acids, and vitamin D [7, 13–15].

The rapid cell differentiation and development of organs make the first trimester a particularly sensitive phase; however, it is a time when the mother might be unaware of her pregnancy [16–20]. Inadequate folic acid intake or excessive vitamin A intake during this phase can induce birth defects [21]. Folic acid is essential for blood formation, neuronal development, and several growth mechanisms [4, 15, 22, 23]. Given the proven efficacy of folic acid supplementation in preventing neural tube defects [24], the DGE recommends that expectant mothers take 400 μg of folic acid daily through dietary supplements. This recommendation is particularly relevant for women starting supplementation one month prior to conception. If the supplementation is initiated at a later stage, then the daily dosage should be adjusted to 800 μg [4, 22].

Iodine is another essential micronutrient during pregnancy, as it is crucial for thyroid hormone synthesis and fetal development [25–27]. A deficiency can lead to severe health complications, including congenital hypothyroidism and cognitive impairment in the fetus [25]. The DGE advises a daily intake of 230 μg of iodine for pregnant women [4]. Approximately 45.9% of German women in the reproductive age group have an inadequate iodine intake, particularly those with low consumption of iodized salt and fish [28]. Consequently, to achieve adequate iodine intake, it is generally recommended to incorporate a daily supplement within the dosage range of 100 to 150 μg/d [4, 8, 22].

Furthermore, clinical guidelines advise pregnant women to abstain from consuming raw or undercooked meat, fish, eggs, and milk due to the potential presence of harmful bacteria, such as Listeria, Salmonella, and Campylobacter, or parasites like *Toxoplasma gondii* [22]. These pathogens can pass through the placenta and result in severe health complications, including miscarriages, neonatal meningitis, sepsis, premature birth, and stillbirth [29].

Moreover, alcohol consumption is strongly discouraged due to the potential for fetal alcohol spectrum disorders [4]. The most severe manifestation within this spectrum is fetal alcohol syndrome, which can result in developmental delays, learning and behavioral issues, facial deformities, motor difficulties, and emotional regulation issues [30]. Pregnant women are also advised to abstain entirely from smoking (including passive smoke) and all forms of nicotine [4], as they can increase the risk of severe deformities, growth delays, and complications such as premature birth or miscarriage [31]. Women are no longer advised to gradually quit or continue smoking at the onset of pregnancy. Even quitting in the third trimester can reduce risks up to birth [32, 33].

Caffeine consumption during pregnancy, however, remains controversial. While a daily caffeine intake of 200 mg is considered safe [34], higher doses have been associated with an increased risk of miscarriages and reduced birth weights [35]. During dietary consultations, it is necessary to educate pregnant women that not only coffee but also black and green tea, mate tea, and energy drinks contain caffeine. For health reasons, the German Nutrition Society (DGE) recommends completely avoiding energy drinks during pregnancy [4].

However, restrictions should be limited to high-risk foods and substances. Overly restrictive eating behaviors are counterproductive [36–39]. Contrary to popular belief, the avoidance of specific foods to reduce the child’s allergy risk is not backed up by evidence [40, 41]. Instead, expectant mothers should follow a well-balanced diet that contains all essential nutrients [42, 43].

Overall, evidence indicates that dietary practices during pregnancy profoundly affect fetal development and the child’s long-term health outcomes. Despite this, recent studies reveal persistent gaps in adherence to nutritional recommendations. In Germany, pregnant women show a higher prevalence of vitamin D deficiency compared with nonpregnant women, particularly during the third trimester and in winter months [44]. Furthermore, a majority of pregnant women across Europe do not meet the recommended intake levels for iodine, folic acid, and iron [45–48]. Collectively, these findings suggest that pregnant women in Germany may not be meeting their nutritional requirements. Therefore, more extensive research is warranted to achieve a thorough understanding of the current dietary patterns of pregnant women in Germany and to develop targeted interventions for enhancing their nutritional status.

Moreover, no recent studies have examined the sources through which pregnant women in Germany acquire nutritional information. Possible sources encompass guidance from healthcare professionals (HCPs), such as gynecologists and midwives, and online resources. In addition, women frequently embrace complementary and alternative medicine for vitamins, herbal supplements, and nutritional counseling, with such services commonly being offered by their HCPs [49].

Despite the growing awareness of the significance of nutrition during pregnancy, a substantial research gap persists in the comprehension of the sources and quality of information on this critical subject. While previous studies have explored specific nutritional aspects relevant to pregnant women in Germany, comprehensive data on the diverse array of information channels used by pregnant women for dietary guidance are still missing. Therefore, we aimed to investigate the dietary habits and recommendations of pregnant women in Germany through an online survey.

## Methods

### Study design

This study employed a noninterventional cross-sectional study design. The data were collected through an online survey in the German language. A combination of inductive and deductive approaches was used to investigate where pregnant women obtain dietary recommendations and whether these recommendations are consistent with the current guidelines of the DGE and the Germany-wide Healthy Start Network of the Federal Center for Nutrition. The design of the questionnaire, conduct of the survey, and data collection were based on the established methodology of empirical research [50].

The questionnaire outcome variables aimed to identify sources of nutritional advice for pregnant women and to assess their alignment with the established guidelines. Furthermore, the questionnaire survey considered the advice provided by HCPs, the sources that participants sought information from, and the accuracy of the information, focusing on areas such as avoided foods, allergy prevention, and dietary supplements. Additionally, the survey evaluated characteristics, such as socioeconomic factors, the depth of nutritional guidance, and the respondent’s focus on nutrition, to determine their influence on the quality and accuracy of the advice provided. Statistical analyses were then conducted to determine the relationship between these predictor variables and the outcome variables.

### Data collection

The inclusion criteria required participants to reside in Germany, be pregnant at the time of the survey with a minimum gestational age of six weeks and have internet access to complete the online questionnaire. Recruitment was conducted via social media platforms, primarily Instagram and other channels focusing on pregnancy and family life. Consecutive sampling was selected as the sampling strategy. It was conducted over 162 days, from January 19 to June 29, 2022. Data were collected using the online tool SurveyMonkey. Prior to participation, all respondents provided written informed consent and were informed that their responses would be anonymized and treated according to German data protection standards. The questionnaire comprised 37 questions (Appendix 1). Participants were instructed to specify the micronutrients contained in their supplements and, with the supplement packaging at hand during survey completion, to record the daily dosage directly from the label to minimize reporting inaccuracies. Standardized scales and previously validated questions ensured the reliability and validity of the results [51]. The participants were asked about their height and weight before pregnancy, and their body mass index (BMI) was calculated based on this information. A person’s BMI was calculated from the square of their height in meters divided by their body weight in kilograms [52].

### Data analysis

Descriptive statistics were applied using R version 4.3.2 to characterize the study population and to indicate the percentage distribution of responses in the questionnaire. The data were subsequently analyzed to determine their alignment with guidelines provided by the Healthy Start Network and the DGE [4, 22], which provide specific recommendations regarding foods to avoid and supplementation during pregnancy. The participants’ responses were categorized as either guideline-congruent or noncongruent.

To determine associations between participant characteristics and the outcomes of interest (e.g., dietary restrictions, supplement use), we employed a combination of bivariate and multivariable analyses. Initially, chi-square tests and t-tests were performed in R to identify significant subgroup differences within the sample; *p* values ≤ 0.05 were considered statistically significant. To address multiple testing, the Benjamini–Hochberg procedure was applied to control the false discovery rate at 0.05 [53].

Regression analyses were performed in R to estimate associations while adjusting for potential confounders. Multivariable logistic regression was used for binary outcomes, and multinomial logistic regression for outcomes with more than two categories. Predictor variables were selected a priori based on theoretical relevance and previous literature, including sociodemographic (e.g., income, educational attainment) and behavioral factors (e.g., nutritional counseling, physical activity, self-information-seeking). Continuous predictors were modeled as originally coded. Results are reported as odds ratios (ORs) with 95% confidence intervals (CIs).

Prior to regression, certain response options were grouped into categories or dichotomies. Income was categorized into two classifications, namely below €2,500 and above €2,500 net. This categorization was chosen based on the OECD equivalence scale, which sets the poverty line for a family with young children at €2,410 [54]. Furthermore, educational qualifications were also divided into academics and non-academics. Participants who did not provide information for a given variable (e.g. specific dosage) were excluded from the respective analyses.

## Results

Out of the 4,351 women who began the survey, a total of 3,363 successfully completed it; thus, the completion rate was 77.00%. While 52.38% of the participants held academic qualifications, indicating a higher educational level than the average population, other characteristics such as age, BMI, and income closely mirrored the average German pregnant woman; thus, the results of the sample are deemed representative of the German pregnant population, with further details provided in Appendix 2 [55–57].

### Sources of dietary recommendations

#### Consultation by healthcare professionals

All 3,363 participants were asked about the extent to which they were advised by HCPs on nutrition and/or dietary supplements during their pregnancy. 52.10% (*n* = 1,031) of the respondents reported receiving “little” advice on nutrition during pregnancy, while 30.66% (*n* = 1,031) reported receiving no advice at all. By contrast, 13.74% (*n* = 462) of the participants reported receiving comprehensive advice, while only 3.51% (*n* = 118) reported receiving very comprehensive advice.

In summary, a majority of the pregnant women (> 80%) received little to no advice. At the same time, 68.66% (*n* = 2309) of the participants reported improving their diet since the beginning of their pregnancy compared to before. Thus, while most women did not receive professional guidance, two-thirds made changes to their dietary habits. Receiving (very) comprehensive advice was significantly correlated with other healthy lifestyle decisions. For instance, these women were more likely to exercise regularly during their pregnancy in the bivariate analysis (*p* < 0.001); however, in regression modeling this association was not statistically significant (OR = 1.08, 95% CI 0.90–1.30, *p* = 0.389). A consistent and significant effect was observed for dietary behavior: women receiving comprehensive advice more frequently reported paying attention to a healthy diet during pregnancy (OR = 1.59, 95% CI 1.24–2.06, *p* < 0.001). Additionally, a correlation was observed between the comprehensiveness of advice and reduced alcohol and nicotine consumption during pregnancy (both *p* < 0.001).

**Fig. 1:**
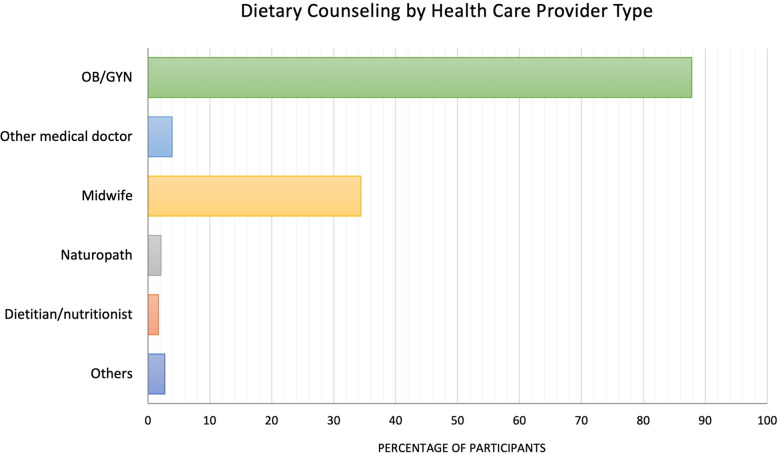
Proportion of participants (*n* = 2,333) receiving dietary counseling from different health care provider types. Multiple responses were possible

When participants indicated that they had received counseling from their HCP, they were requested to identify the specific provider. In response, 87.78% (*n* = 2,048) of respondents stated that the advice had come from an obstetrician or gynecologist. Midwives were the next most common source of dietary advice (34.33%, *n* = 801). Other medical professionals were comparatively less represented (Fig. 1). Within the “Others” category (*n* = 64), pharmacists were frequently cited; Only 1.69% (*n* = 57) of the pregnant women received advice from a nutrition specialist. Figure 1 presents the healthcare professionals providing dietary counseling within the sample.

#### Participants’ initiative in seeking nutritional information

Participants were also asked whether they had personally taken the initiative to inform themselves about nutrition and dietary supplements, which 80.79% (*n* = 2,717) of the women answered in the affirmative. A link between guidance from HCPs and personal initiative in seeking nutritional information was observed: Of those 118 women who received very comprehensive advice, 84.70% (*n* = 100) took the initiative to inform themselves further. This trend was consistent for women who received detailed (86.6%) or little advice (84.00%). Conversely, among women who received no advice from HCPs, only 72.20% had made the effort to inform themselves (*p* = 0.034).

#### Sources of information

**Fig. 2:**
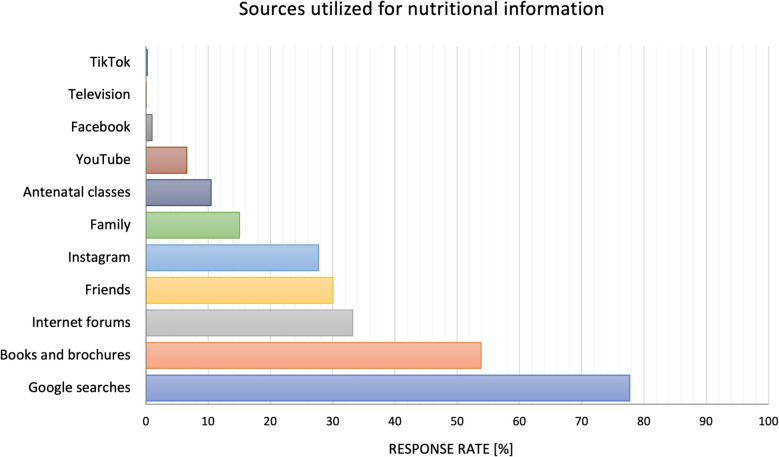
Various online and offline sources used by the participants to obtain nutritional information. Multiple responses were allowed. Percentages are relative to the total sample (*n* = 3,363)

Next, the participants were asked to identify their top three sources of information. As depicted in Fig. 2, 77.70% (*n* = 2,111) of the pregnant women primarily relied on Google searches for nutritional insights. Books and brochures followed as the second most frequently consulted source (53.81%, *n* = 1,462), while Internet forums were the third most popular source (33.24%, *n* = 903). Other sources of dietary information included friends and acquaintances (30.03%, *n* = 816), family members (15.02%, *n* = 408), and childbirth preparation courses (10.45%, *n* = 284). Moreover, 27.71% (*n* = 753) of the women turned to Instagram for guidance. By contrast, other social media platforms such as YouTube (6.55%, *n* = 178), Facebook (0.99%, *n* = 27), and TikTok (0.22%, *n* = 6) were used considerably less frequently. Figure 2 presents online and offline sources that were utilized by the participants to seek nutritional information.

### Information quality and guideline conformity

#### Avoidance of foods

Participants were asked about the foods that they were advised to avoid and were given a list of 31 food items. Of these, current guidelines suggest that only nine are genuinely contraindicated during pregnancy [4, 22]. A significant proportion of participants indicated that they had received accurate advice regarding foods that pose potential health risks. Specifically, 95.33% (*n* = 3,206) were cautioned against consuming raw meat, 89.18% (*n* = 2,999) against unpasteurized milk and dairy, 92.42% (*n* = 3,108) against raw fish and seafood, and 85.16% (*n* = 2,864) against raw sausages (e.g., salami).

For other foods, however, the recommendations were less consistent. Only 44.37% (*n* = 1,492) of the participants were informed about the risks associated with wild game and offal, and a mere 25.33% (*n* = 852) were advised against beverages that contain quinine, such as tonic water and bitter lemon. The consensus was stronger regarding alcohol and nicotine, with 96.73% (*n* = 3,253) and 96.37% (*n* = 3,241) as well as Energy drinks with 72.79% (*n* = 2,448).

Noteworthily, some recommendations extended to foods that are not inherently hazardous during pregnancy. For instance, 31.22% (*n* = 1,050) were mistakenly cautioned against consuming coffee and 28.55% (*n* = 960) against consuming black tea. Furthermore, 19.30% (*n* = 649) were advised to abstain from sugar consumption, 16.06% (*n* = 540) received warnings about specific spices (e.g., cinnamon, cardamom, and cloves). Further items were honey (9.10%, *n* = 306), poppy seeds (9.66%, *n* = 325), and white flour products (13.53%, *n* = 455).

To examine these foods in more detail, we separately evaluated the number of foods avoided in alignment with the guidelines (termed “evidence-based avoidances”) and those avoided contrary to the guidelines (termed “non-evidence-based avoidances”).

##### Evidence-based avoidances

On average, expectant mothers were counseled to steer clear of almost seven (6.98; SD ± 1.53) of the nine foods that the guidelines recommend avoiding. There was no statistically significant difference based on educational levels (*p* = 0.06) or income levels (*p* = 0.08).

**Fig. 3:**
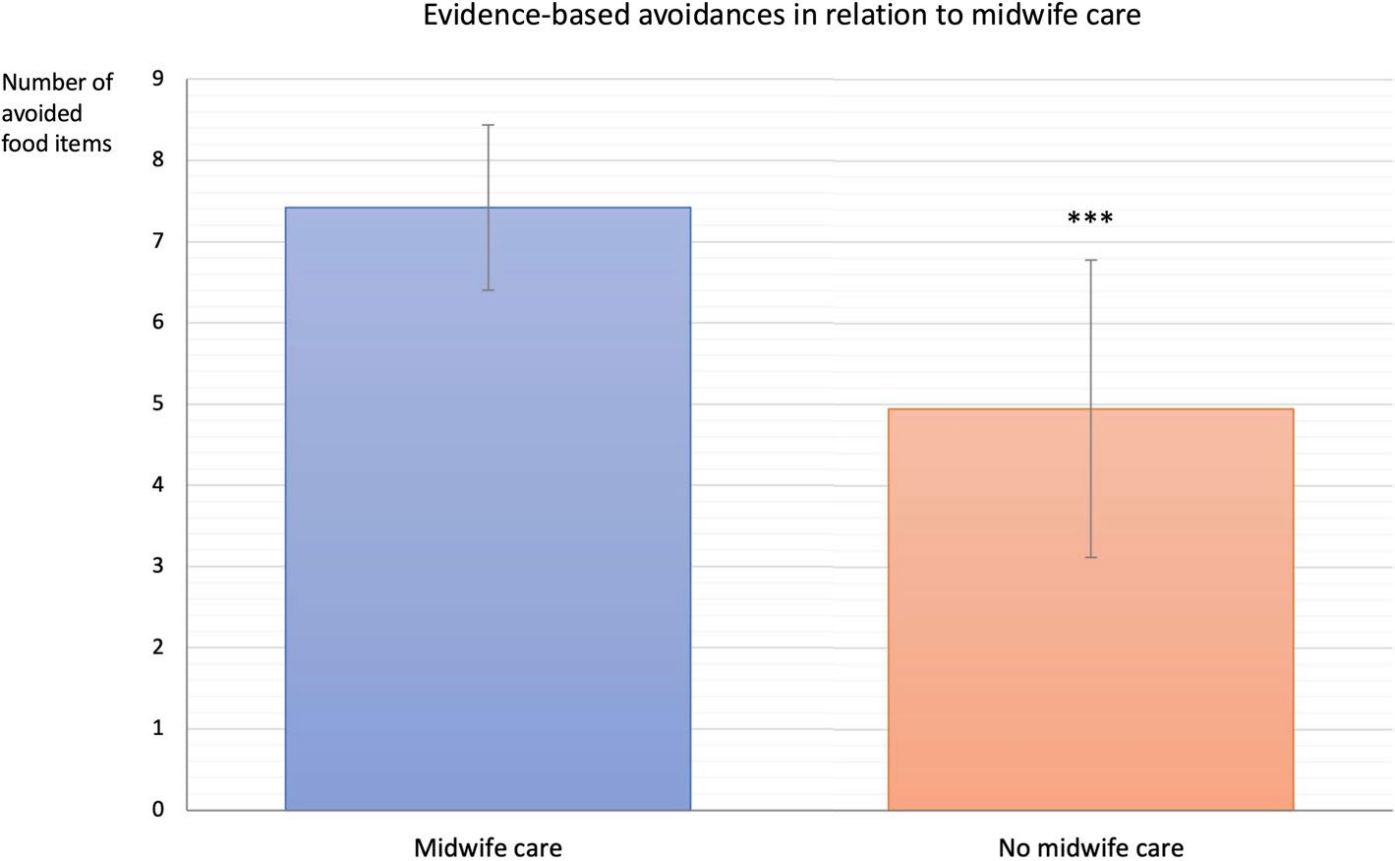
Number of evidence-based avoided foods among participants with (*n* = 2762) and without (*n* = 601) midwife care. Participants receiving midwife care showed significantly more evidence-based avoidance than those without (*p* < 0.001)

Furthermore, women who placed a higher emphasis nutrition during pregnancy or received comprehensive dietary counseling from HCPs were more accurately informed (7.03 vs 6.75, *p* < 0.001; 7.25 vs 6.93, *p* < 0.001, respectively). Midwife care was associated with the highest adherence (7.42 vs 4.95, *p* < 0.001), and self-informed participants also avoided more foods than those who did not (7.08 vs 6.54, *p* < 0.001; Fig. 3). Thorough details on this analysis can be found in the supplementary material (appendix 3). Figure 3 presents the number of evidence-based avoided foods in correlation to midwife care.

##### Non-evidence-based avoidances

**Fig. 4:**
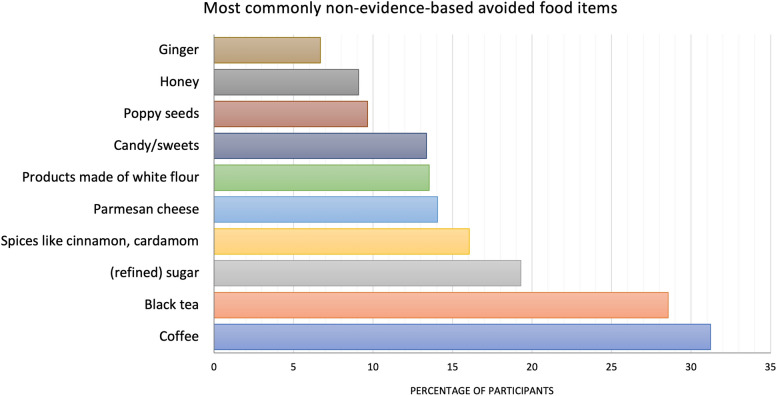
Percentage of participants avoiding foods not recommended for complete abstinence according to guidelines (*n =* 3,363). Multiple responses were possible

In general, coffee was the food item mentioned the most (31.22%, *n* = 1,050), followed by black tea (28.55%, *n* = 960) and (refined) sugar (19.30%, *n* = 649). Figure 4 presents the 10 most frequently non-evidence-based avoided foods:

On average, the participants reported avoiding 1.88 (SD ± 2.1) foods that the guidelines do not explicitly caution against. In total, 20 foods on the provided list were not deemed necessary to completely avoid. Similar to the previous category, no significant differences were observed by education (academics: 1.93 vs non-academics: 1.83; *p* = 0.17) or income (below €2,500: 1.87 vs above €2,500: 1.89; *p* = 0.87). In contrast, women who prioritized a health-conscious diet, received comprehensive counseling, had midwife care, or self-informed about nutrition tended to avoid more non-evidence-based foods (1.96 vs 1.56; 2.07 vs 1.82; 1.99 vs 1.36; 2.04 vs 1.23; all *p* < 0.01).

#### Avoidance of foods for allergy prevention

Within the pregnancy cohort, women were questioned about food avoidance with the intention of allergy prevention. 25.48% (*n* = 857) of all participants indicated they had been advised or encountered this respective information during their self-informed endeavors.

**Fig. 5:**
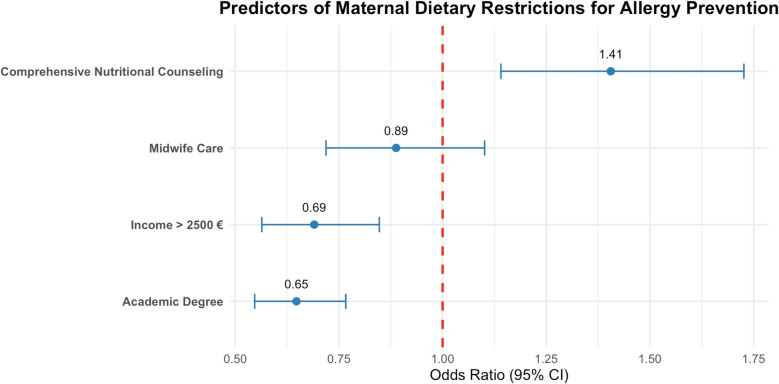
Multivariable associations of sociodemographic and counseling factors with maternal dietary restrictions for allergy prevention. ORs and 95% CIs are presented (*n* = 3,363)

This misinformation was associated with educational background and household income levels (see Fig. 5). Participants with higher income had a 31% lower likelihood of following such restrictions (OR = 0.69, 95% CI 0.56–0.85), and individuals with an academic degree a 35% reduced likelihood (OR = 0.65, 95% CI 0.55–0.77; *p* < 0.001). In contrast, women who received very comprehensive counseling from healthcare practitioners showed a pronounced inclination toward allergy prevention advice (39.83%, OR = 1.41, 95% CI 1.14–1.73; *p* < 0.001). The effect of midwife care remained nonsignificant (OR = 0.89, 95% CI 0.72–1.10; *p* = 0.07).

#### Intake of dietary supplements

Regarding the intake of dietary supplements during pregnancy, 89.03% of respondents (*n* = 2,994) reported either currently taking supplements or having taken them at some point during their pregnancy, while 10.97% (*n* = 369) did not.

**Fig. 6:**
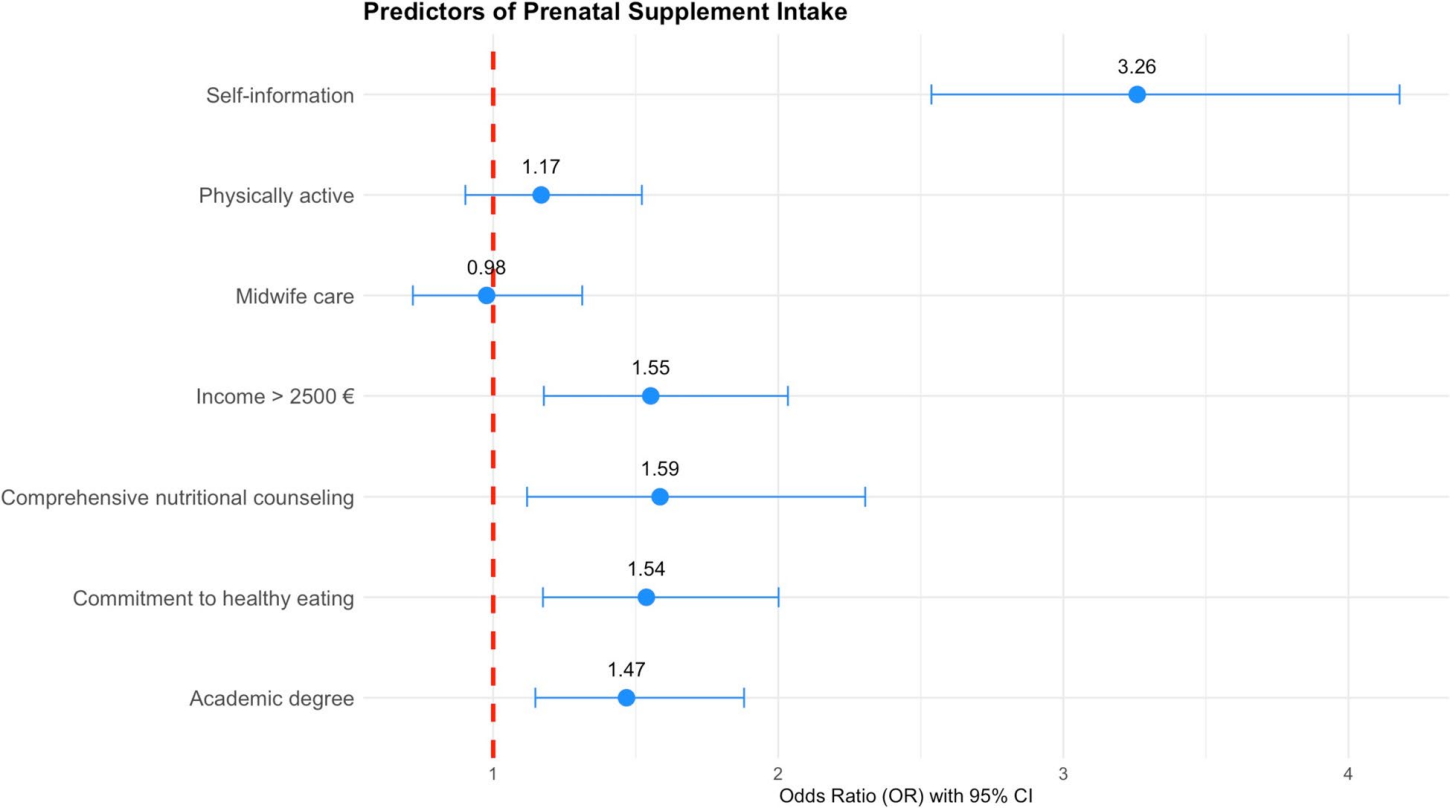
Sociodemographic, behavioral, and counseling predictors of prenatal supplement intake. Results are presented as odds ratios (ORs) with 95% confidence intervals (CIs) (*n* = 3,363)

Higher household income (> €2,500) was associated with 55% higher odds of supplement intake (OR = 1.55, 95% CI 1.18–2.03, *p* < 0.01), and women with an academic degree had 47% higher odds compared with those without (OR = 1.47, 95% CI 1.15–1.88, *p* < 0.01). Strong commitment to healthy eating was also a significant predictor (OR = 1.54, 95% CI 1.17–2.00, *p* < 0.01), as was receiving comprehensive nutritional counseling (OR = 1.59, 95% CI 1.12–2.31, *p* < 0.05). Self-initiated information seeking was the strongest predictor, tripling the odds of supplement use (OR = 3.26, 95% CI 2.54–4.18, *p* < 0.001). In contrast, alcohol consumption and smoking showed no significant influence (*p* = 0.86 and *p* = 0.26, respectively). Regular physical activity (OR = 1.17, 95% CI 0.90–1.52, *p* = 0.24) and midwife care (OR = 0.98, 95% CI 0.72–1.31, *p* = 0.88) were also not significantly associated with supplement intake (Fig. 6).

##### Intake of folic acid

Participants were instructed to specify the micronutrients in their supplements and to report the daily dosage directly from the packaging. Of the women who took dietary supplements (*n* = 2,994), 98.70% (*n* = 2,955) included folic acid, equating to 87.87% of all participants. This indicates that 408 women abstained from folic acid intake, contrary to guideline recommendations. The advised dosage varies depending on the time of initiation: 400 μg daily if commenced prior to pregnancy, or 800 μg upon the onset of pregnancy [4, 22]. However, a subset (*n* = 804) did not provide information on dosage or timing.

**Fig. 7:**
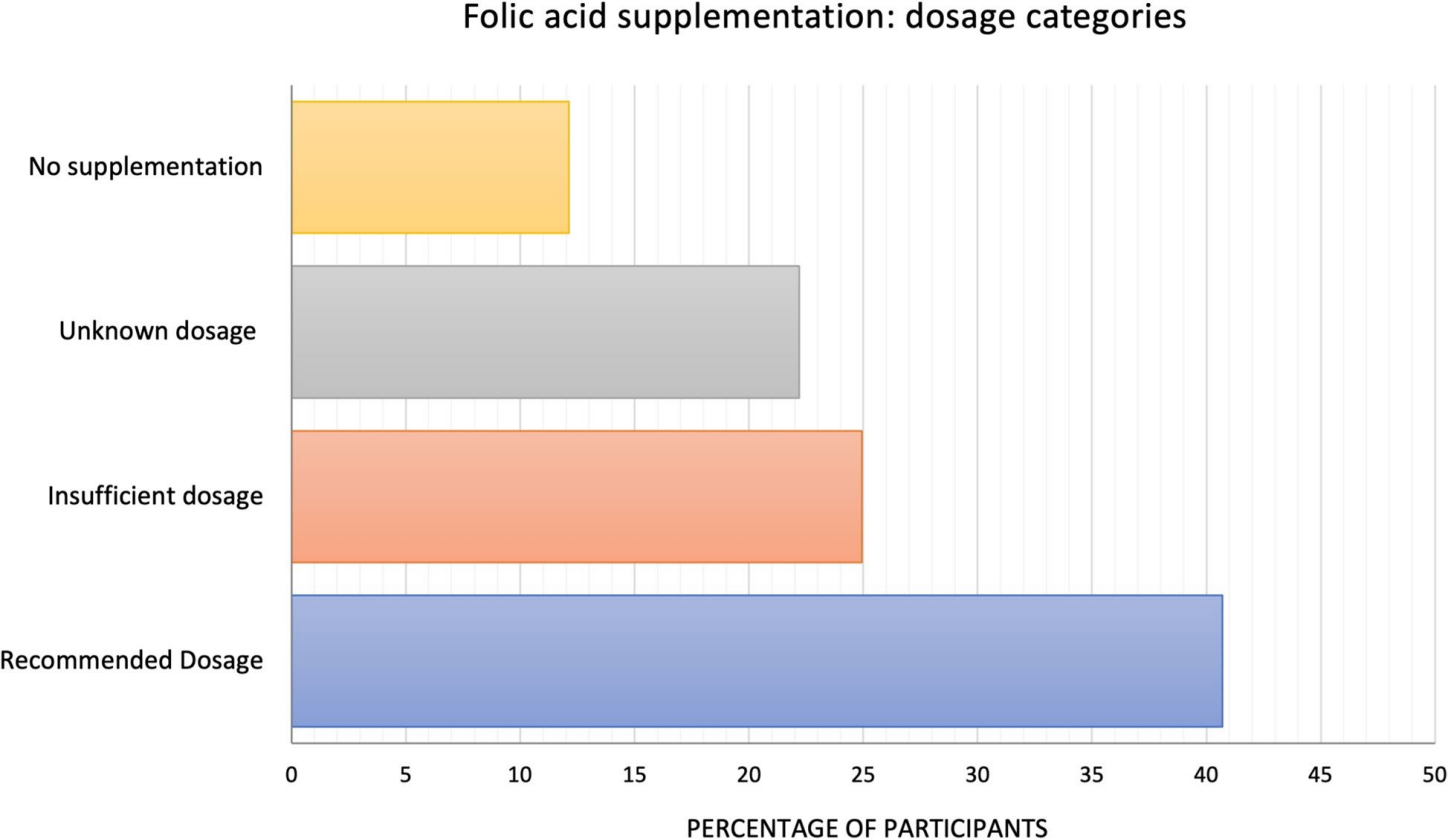
Percentage of participants supplementing with folic acid, categorized by supplementation status: no supplementation (*n* = 408), unknown dosage (*n* = 747), insufficient dosage (*n* = 839), and recommended dosage (*n* = 1,369)

Overall, 40.71% (*n* = 1,369) of women adhered to the recommended dosage, 24.95% (*n* = 839) reported suboptimal intake, 22.21% (*n* = 747) supplemented without specifying dosage, and 12.13% (*n* = 408) did not supplement at all. This yields a conservative estimate that at least 40.71% met recommendations, while a minimum of 37.08% (*n* = 1,247) had insufficient intake. Figure 7 illustrates the distribution of folic acid supplementation within the sample.

**Fig. 8:**
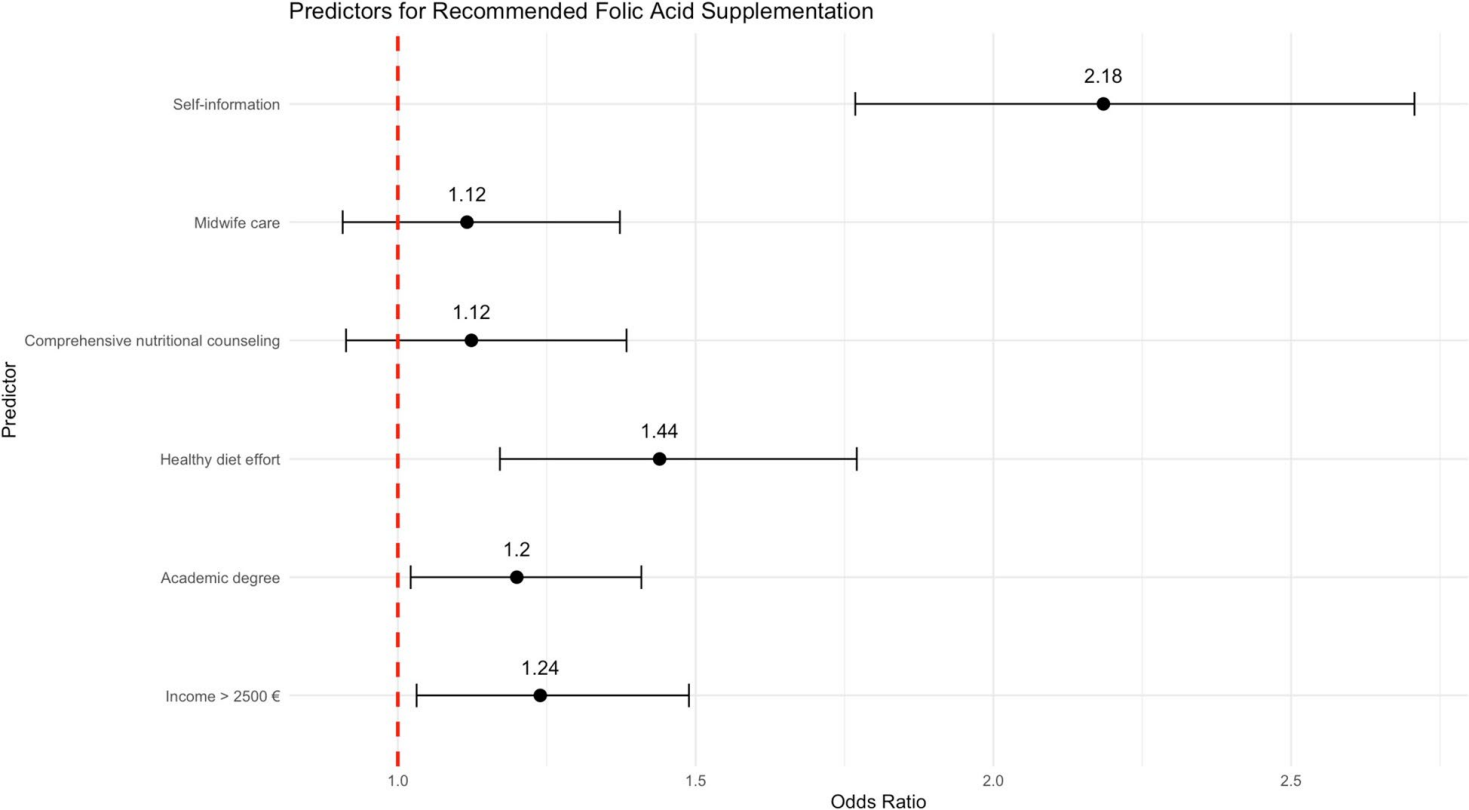
Sociodemographic, behavioral, and counseling predictors of recommended folic acid intake during pregnancy. Results are presented as odds ratios (ORs) with 95% confidence intervals (CIs) (*n* = 3,363)

Several factors were associated with folic acid supplementation. Women with higher household income (> €2,500) had greater odds of intake (OR = 0.67, 95% CI 0.53–0.84, *p* < 0.001), as did those who reported strong efforts toward a healthy diet (OR = 0.75, 95% CI 0.61–0.92, *p* = 0.005). Self-information was the strongest predictor, substantially increasing the likelihood of intake (OR = 0.54, 95% CI 0.43–0.68, *p* < 0.001). Academic education (OR = 0.84, 95% CI 0.70–1.01, *p* = 0.06), comprehensive counseling (OR = 0.87, 95% CI 0.70–1.08, *p* = 0.21), and midwife care (OR = 0.88, 95% CI 0.72–1.07, *p* = 0.19) showed no significant associations (Fig. 8).

Most women who supplemented with folic acid reported gynecologists as their primary source of advice (87.44%, *n* = 2,584), followed by midwives (27.72%, *n* = 819). Online information (26.76%, *n* = 791) and recommendations from family, friends, and peers (24.16%, *n* = 714) were also relevant sources.

##### Intake of iodine

**Fig. 9:**
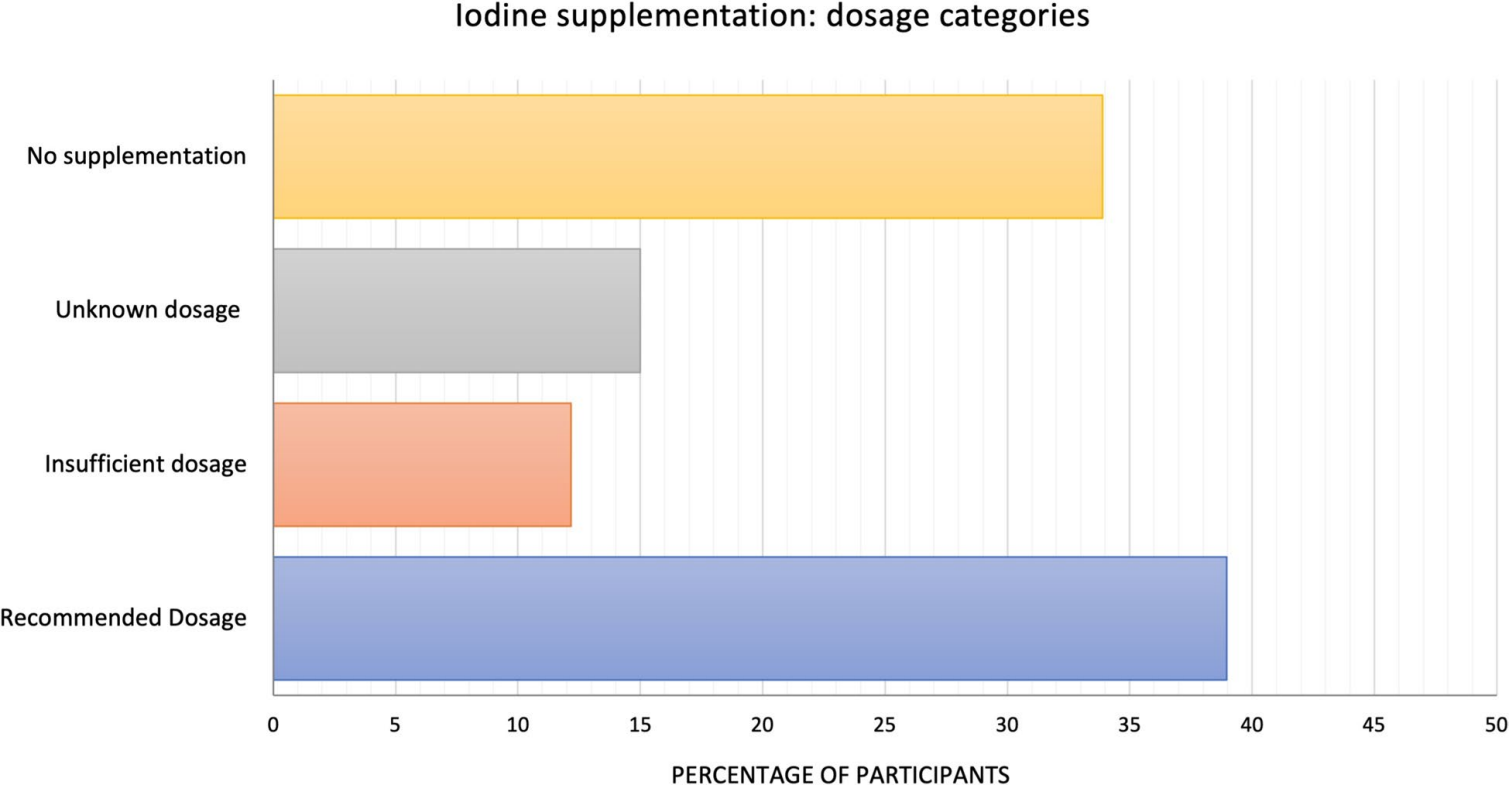
Percentage of participants supplementing with iodine, categorized by supplementation status: no supplementation (*n* = 1,140), unknown dosage (*n* = 504), insufficient dosage (*n* = 409), and recommended dosage (*n* = 1,310)

Furthermore, participants reported whether they supplemented with iodine and, if applicable, specified their dosage. The primary source of the recommendation for iodine intake was their gynecologist, as noted by 1,434 participants. Overall, 66.10% (*n* = 2,223) of women reported taking iodine supplements, while 33.90% (*n* = 1,140) did not. Among those supplementing, 77.32% (*n* = 1,719) provided their specific dosage. Of these, 38.95% (*n* = 1,310) adhered to the recommended amount, whereas 12.16% (*n* = 409) reported suboptimal intake. The remaining 504 women (14.99%) supplemented without specifying their dosage, preventing exact evaluation (see Fig. 9). Taken together, these data allow a conservative estimate: at least 38.95% of participants supplemented with the recommended dosage, while insufficient intake is confirmed for the 224 women with suboptimal intake plus the 1,140 women who did not supplement at all. Conservatively combining these subgroups yields a minimum of 46.06% (*n* = 1,549) of women with inadequate iodine intake.

Comprehensiveness of professional counseling was strongly associated with iodine intake. Women who received comprehensive advice were more likely to supplement compared with those who received minimal or no guidance (*p* = 0.001). Notably, only 33.9% (*n* = 97) of women who neither received advice nor informed themselves took iodine, while supplementation was most frequent among women who both received professional advice and engaged in self-information (77.1%, *p* < 0.001). Midwife or nutrition specialist care was also associated with higher supplementation rates (both *p* = 0.001).

**Fig. 10:**
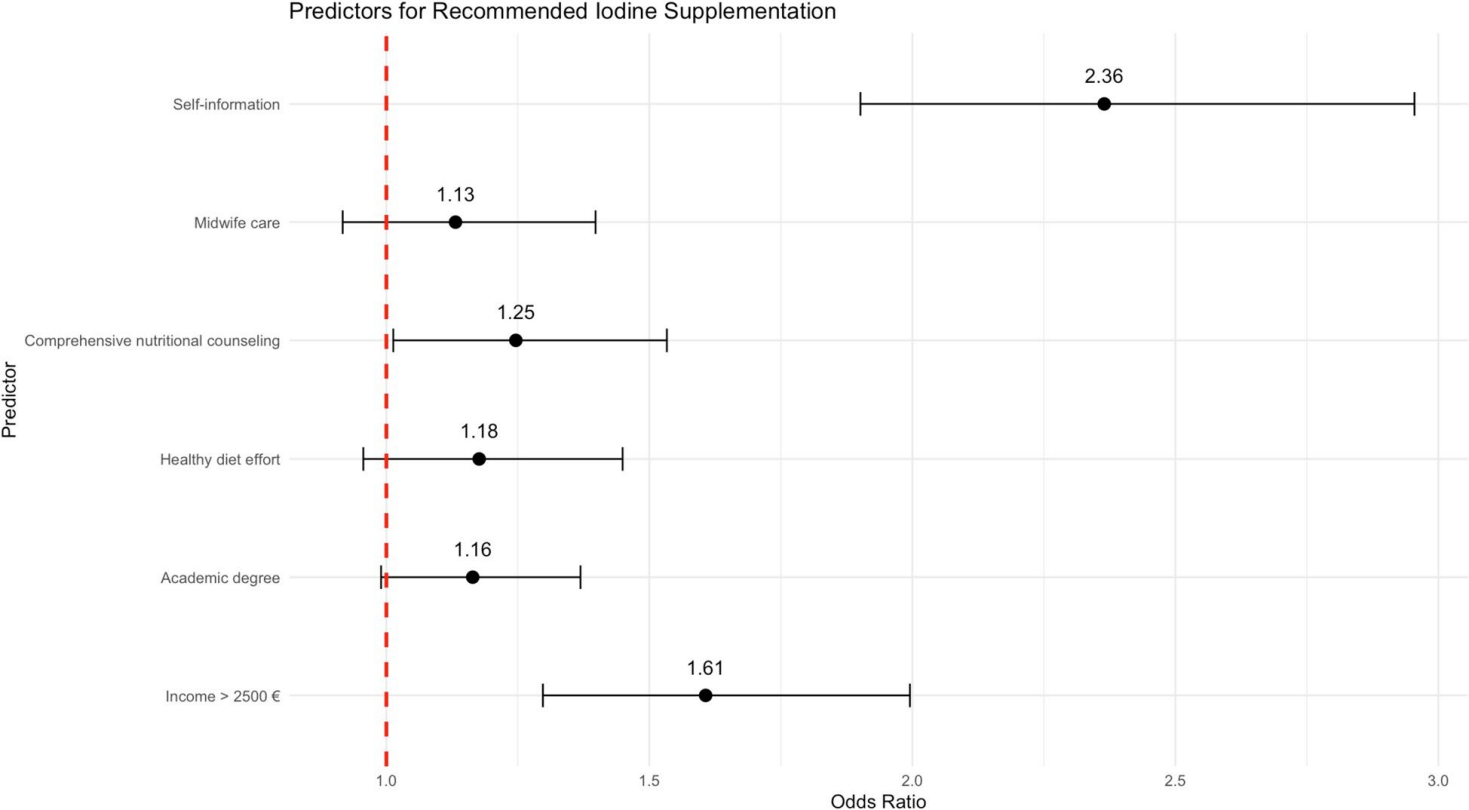
Factors associated with iodine supplement intake in pregnancy. Results are presented as odds ratios (ORs) with 95% confidence intervals (CIs) (*n* = 3,363)

Binary logistic regression further highlighted that higher household income (> €2500; OR = 1.61, 95% CI [1.30–2.00], *p* < 0.001), comprehensive nutritional counseling (OR = 1.25, 95% CI [1.01–1.53], *p* = 0.037), and active self-information (OR = 2.36, 95% CI [1.90–2.95], *p* < 0.001) were significantly associated with iodine supplementation. In contrast, academic degree (OR = 1.16, 95% CI [0.99–1.37], *p* = 0.066), emphasis on a healthy diet (OR = 1.18, 95% CI [0.96–1.45], *p* = 0.125), and midwife care (OR = 1.13, 95% CI [0.92–1.40], *p* = 0.250) showed no statistically significant associations (Fig. 10).

## Discussion

Knowledge of healthy nutrition during pregnancy is crucial for pregnant women, as it directly influences the development of the unborn child and lays the foundation for a healthy life. This comprehensive study, involving 3,363 pregnant women in Germany, revealed that nutrition is of great interest to most pregnant women, which points to the need for increased nutritional counseling and informational support. The findings indicated that a considerable fraction (81%) of respondents paid more attention to healthy eating during pregnancy; as a result, nearly 69% had made efforts to improve their diet upon the onset of their pregnancy. Consequently, many of these women proactively searched for nutritional information from various sources. The following discussion will cover the role of healthcare professionals, alternative information sources, dietary advice, supplements, study limitations, strengths, and future research insights.

### Role of HCPS in nutritional counseling

Medical professionals, particularly gynecologists and midwives, often serve as the primary sources of information for expectant mothers. However, this study highlights a potential deficiency in the counseling of nutritional information. A significant percentage – 82.76% – of respondents reported receiving little to no nutritional advice during their prenatal consultations. This gap suggests the need for policy measures to integrate standardized nutritional counseling into routine prenatal care to reduce health disparities and optimize pregnancy outcomes. In general, the involvement of nutritionists and dietitians seemed limited. Nutritional counseling by a nutritionist is not standard care in Germany and is mostly not reimbursed by health insurance, which may limit its use among pregnant women. These findings are not limited to Germany, as they have also been reported in many other countries, such as the United States [58], Canada [59], the United Kingdom [60] and the Netherlands [61]. General nutritional guidance may suffice for most pregnancies without complications. However, in specific conditions such as diabetes and obesity, a consultation with a nutritionist is highly recommended [62, 63]. This is particularly relevant considering the incidence of GDM, which affects up to 14% of pregnant women in Germany [64, 65]. Other medical conditions, such as diabetes mellitus type 2, obesity, preeclampsia, and irritable bowel syndrome, could also benefit from a consultation with a nutritionist [63, 66–68]. In conclusion, while there is a clear interest in healthy nutrition among pregnant women in Germany, there remains a gap in the provision of detailed and specialized nutritional guidance.

### Other information sources

While medical professionals remain a primary source of information, over 80% of pregnant women also seek guidance from other sources. Google stands out as the predominant platform, with nearly 78% of respondents turning to it for answers. This trend aligns with the broader population’s behavior, especially among young women [69] in Germany. It also appears to mirror the preferences of pregnant individuals and parents globally, aligning with a general worldwide trend [58, 70–72]. Other sources include internet forums, social media, books, and advice from family and friends. It is crucial to note that the quality of information available online varies significantly, with many search results lacking evidence-based advice [73]. From a public health perspective, it would be highly desirable for these alternative channels to provide reliable, evidence-based information. However, in practice, achieving this remains challenging, as misinformation is widespread on social media and difficult to regulate or fully control. Some official institutes and professional societies offer high-quality, evidence-based information, but their accessibility and comprehensibility for the public remain uncertain. Similarly, recommendations from friends and family members, cited by one-third of the participants, can be problematic. While social media platforms have emerged as information sources, they often propagate misleading or incorrect advice [74–76].

Interestingly, women who actively sought additional information were also more likely to report receiving comprehensive advice, suggesting that nutritionally motivated individuals tend to combine professional guidance with independent research. This pattern is consistent with previous studies that have indicated that information seeking (also for nonpregnant people) from HCPs, media, and family/friends increases the likelihood of engaging in healthy lifestyle behaviors and changing one’s diet [77], which was also observed for pregnant women [78]. Conversely, women who received limited or no consultation were less inclined to supplement adequately, which may reflect both a lower awareness of nutritional needs and a generally lower health motivation. At the same time, our findings suggest that professional counseling alone was less strongly associated with supplement intake than self-information, possibly because motivated women are more likely to both seek advice and actively research on their own. This highlights a potential confounding effect of individual health orientation, rather than an inherent ineffectiveness of counseling. From a public health perspective, these results underline the importance of improving the accessibility, clarity, and consistency of professional nutritional counseling, while simultaneously ensuring that evidence-based resources are more visible and user-friendly.

### Guideline adherence

#### Avoidance of potentially critical foods

This study also examined the avoidance of foods or substances that could potentially be harmful during pregnancy. Abstaining from raw animal products, alcohol, and nicotine have become broadly accepted practices. Remarkably, participants refrained on average from almost seven of the nine potential items listed in the survey. Yet, this raises concerns that 2.02% of the respondents acknowledged occasional alcohol consumption during their pregnancy, which hints that the actual number might be higher. Notably, 84.70% of the German population reports some level of alcohol consumption [79], and specific investigations focusing on pregnant women in Germany revealed that 27.60% may partake in alcohol consumption during pregnancy, as indicated by fetal alcohol syndrome statistics [80]. In a comparative context, European countries exhibit higher rates of alcohol consumption during pregnancy than the global average [81]. This phenomenon could be attributed to cultural differences and varying societal norms regarding the significance of alcohol. Europe, in general, boasts some of the world’s highest per capita alcohol consumption levels, with Germany exceeding the European regional average [82, 83]. This emphasizes the pressing need for more robust awareness initiatives regarding the implications of alcohol intake during pregnancy, particularly given that the reported reasons for such behavior are often rooted in societal pressure and the misconception that small quantities pose no harm [84].

#### Overly restrictive dietary advice

Pregnant women are sometimes mistakenly advised to follow overly restrictive diets, including the elimination of sugar, coffee, black tea, or certain spices. While moderation may be reasonable, total avoidance is not evidence-based. Notably, one quarter of participants reported recommendations to exclude specific foods to prevent allergies in the child, despite clear evidence to the contrary[4, 22, 85]. Such misinformation appeared more common among women with lower socioeconomic status, while more extensive counseling was paradoxically linked to a higher likelihood of restrictive advice, indicating that misconceptions may sometimes be transmitted even within professional settings. This highlights persisting inconsistencies in nutritional counseling, a challenge also reported in the UK, where midwives often feel uncertain about food avoidance during pregnancy [86]. Beyond professional input, restrictive behaviors may also reflect individual health orientations, as women strongly interested in “healthy eating” can be prone to unnecessary restrictions—a pattern consistent with broader dietary trends [87–89]. It should also be acknowledged that multiple factors may contribute to this association, and it is important to note that the information is based on self-reported data.

### Dietary supplements

According to the guidelines, pregnant women are recommended to take dietary supplements that contain folic acid and iodine [4, 22]. Nevertheless, approximately 1 in 10 pregnant women abstain from any dietary supplementation. Clear socioeconomic disparities are observed, where women of lower income and educational background are less likely to take supplements. These tendencies can also be observed among pregnant women in other European countries and the USA [90–93]. Both comprehensive nutritional counseling and proactive self-information were associated with higher supplement use, with self-information being the strongest predictor. Nearly all women advised by a nutrition expert adhered to recommendations. Considering public health objectives, implementing structured educational programs alongside policies that subsidize or otherwise facilitate access to these key supplements could help reduce socioeconomic disparities in adherence, promoting equitable nutritional support for all pregnant women. Concretely, this could involve health insurance coverage for essential supplements and enabling healthcare providers to prescribe or recommend them during routine prenatal visits.

#### Folic acid intake

Many women reported taking folic acid supplements during pregnancy; however, among those who specified their dosage, less than 40% met the recommended intake, while nearly a quarter reported suboptimal intake or no supplementation at all. These figures represent a minimum estimate, as 22% of participants did not report their dosage, and actual rates of insufficient supplementation may be higher. There is a clear need for improved guidance, though our findings suggest that independent information-seeking is a stronger predictor of adequate folic acid intake than professional counseling alone. One strategy is to proactively address folic acid during gynecological examinations for women of childbearing age, ensuring timely advice before conception. However, considering that approximately one-third of pregnancies in Germany are unplanned [94], this approach would only reach a fraction of women.

Other countries, such as the USA and Canada, have opted for the widespread fortification of staple foods with folic acid, which led to measurable reductions in NTDs [95–97]. While some studies have raised concerns regarding high folic acid intake and potential cancer risk [98–100], evidence remains conflicting, with other studies even suggesting a protective effect [99, 101]. Considering the relatively low incidence of neural tube defects in Germany (9.1 per 10,000 live births [102] and the multifactorial etiology of these defects, a careful benefit–risk assessment is warranted. From a public health perspective, fortification represents an effective and low-barrier strategy to improve folic acid intake across the population [103].

#### Iodine intake

In parallel to folic acid, clinical guidelines also recommend iodine supplementation during pregnancy [4, 22]. Yet, among this study’s participants, only 66% adhered to this recommendation. When excluding participants without reported dosage, the conservative estimate indicates that 46% did not meet the recommended intake; the actual figure is probably higher. Generally, the iodine intake among the public in Germany is suboptimal, particularly due to low consumption of iodized salt and fish, with approximately 45.9% of women in this age group being deficient [28]. A recent survey of gynecologists in Germany revealed that 88.40% are aware of the significance of iodine during pregnancy [104]. This raises the question of why supplementation rates remain low: Is it due to inadequate advice during the limited time in consultations, or are patients not following their recommendations? Our findings highlight the critical role of nutritional education: women who received comprehensive counseling combined with self-information had markedly higher supplementation rates (77.1%), with self-information emerging as the strongest predictor (OR = 2.36). Comprehensive counseling alone also increased adherence (OR = 1.25), whereas midwife care showed no significant effect.

Internationally, similar patterns are observed. In the USA, clinical provision of iodine supplements improved iodine status [105]. In Australia, where mandatory iodine fortification in foods began in 2009, overall iodine intake has increased; however, recommended urinary concentrations are still not met without supplementation. Notably, 81.09% of Australian women adhere to the recommendation of supplementing with iodine during pregnancy, and 62.45% adhere to the recommended dosage [106]. In Portugal, introduction of official recommendations in an iodine-deficient region improved both supplement rates and urinary iodine concentration [107]. These global perspectives emphasize that iodine supplementation must be comprehensively addressed during antenatal consultations to enhance the overall situation.

### Strengths and limitations

A notable strength of this study was its comprehensive data collection. The large sample, reflecting general socioeconomic parameters, allowed for a reliable representation of pregnant women in Germany. While a more detailed evaluation of the accuracy of individual information sources would have been beneficial, most respondents used multiple sources, making it difficult to attribute specific recommendations to a single source.

The survey focused on selected aspects of guidelines. Basic recommendations, such as daily fruit and vegetable intake, and moderate weight gain, are associated with improved pregnancy outcomes [108], but they were not assessed in this survey, as comprehensive dietary assessment (e.g., detailed food group analysis or dietary records) would have exceeded the scope and feasibility of this study. Future research should incorporate detailed dietary evaluation to provide a fuller picture of maternal nutrition.

Other considerations include vegetarian and vegan diets, which are followed by a considerable portion of the population [109] and thus warrant consideration. For these diets, additional supplementation is recommended. While many vegetarians and vegans already supplement with vitamin B12 [110], HCPs should actively inquire and recommend accordingly.

Like all surveys, this study is subject to limitations related to self-reported data. Recall bias and inaccuracies in reporting may have affected responses, particularly for potentially sensitive questions, such as alcohol or nicotine consumption. Additionally, responses requiring participants to remember details from earlier stages of pregnancy, such as the initiation of supplement use, may have been impacted. The recruitment approach, though broad, could have introduced selection bias, reflected in the high proportion of highly educated women and participants with strong interest in nutrition. Higher educational levels are often associated with greater health awareness, including nutritional knowledge and willingness to engage in healthy behaviors. Moreover, our findings that self-information emerged as the strongest predictor of supplement use suggest that this bias may have been amplified. Detailed urban, suburban, or rural data were not collected, limiting assessment of regional disparities. Future studies should investigate urban–rural differences and regional social inequalities, as these factors influence access to healthcare, information, and nutrition behaviors. This consideration is particularly important from a global public health perspective, where disparities in population density and healthcare availability can be more pronounced than in Germany.

## Conclusion

The study aimed to identify the sources pregnant women in Germany use for nutritional advice and to evaluate the alignment of this advice with established clinical guidelines. Notably, only a minority of pregnant women reported received comprehensive nutritional counseling from professionals such as gynecologists, midwives, or nutritionists. A significant number turned to alternative sources for additional guidance, such as the internet, books, friends, and family. While most participants were well-informed about fundamental recommendations, including folic acid supplementation and the avoidance of specific high-risk foods, many of the dietary recommendations they followed did not align with those of professional societies, their dietary practices frequently deviated from established professional guidelines. This was especially evident for iodine supplementation and the adoption of restrictive eating behaviors. Deviations were particularly pronounced among women who neither received professional counseling nor actively sought information themselves. Our analyses highlight that self-information was the strongest predictor of supplement use, emphasizing the critical role of proactive engagement in achieving guideline-consistent behaviors.

These findings underscore the significant role healthcare professionals play in guiding pregnant women. It is crucial that all pregnant women, particularly those less proactive in seeking information, receive clear, evidence-based nutritional advice.. Any misconceptions or inaccuracies they might have encountered should be addressed and rectified. From a public health perspective, improving both the reach and quality of prenatal nutritional counseling could support healthier pregnancies and reduce disparities in maternal and fetal outcomes. Enhancing training for gynecologists and midwives is essential, given their pivotal advisory role. In cases with specific health conditions, such as GDM and preeclampsia, consultations with a nutritionist are desirable. In today’s digital age, where many women rely on online information, official institutes and professional societies should maintain a strong, user-friendly online presence. Digital platforms, including health insurance-supported applications, could help make essential information more accessible and provide ongoing support, particularly when in-person consultations are limited. Overall, there is a clear need for standardized, high-quality dietary counseling to ensure optimal nutrition during pregnancy, prevent overly restrictive dietary practices, and minimize potential risks for both mother and child.

## Supplementary Information

Supplementary Material 1. 

## Data Availability

All data supporting the findings of this study are available within the paper. More comprehensive datasets are available from the corresponding author on reasonable request.

## Abbreviations

BZfE: Bundeszentrum für Ernährung (The Federal Centre for Nutrition, Germany)
BMI: Body mass index
DGE: Deutsche Gesellschaft für Ernährung (German Nutrition Society)
GDM: Gestational diabetes mellitus
HCP: Health care professional
NTD: Neural tube defect
SD: Standard deviation
