# Supplementary material for: Sources of dietary recommendations and adherence to clinical guidelines in pregnant women in Germany
Source: BMC Pregnancy Childbirth. 2025 Oct 13;25:1072. doi: 10.1186/s12884-025-08228-1 (PMC12516835; doi:10.1186/s12884-025-08228-1)
Supplement: Supplementary file 1 — Supplementary Material 1. [file 12884_2025_8228_MOESM1_ESM.docx]

**Appendix 1**

**Survey**

**Pregnancy Details**

**1. Are you currently pregnant?**

- Yes

- No

**2. How many weeks pregnant are you?**

**3. In which week of pregnancy was your pregnancy confirmed?**

**4. Is this your first pregnancy?**

- Yes

- No

**5. Are you receiving care from a midwife during your pregnancy?**

- Yes

- No, I couldn't find a place

- No, I didn't want this

**6. Who has been primarily responsible for your prenatal care so far?**

- Gynecologist

- Other specialist (e.g., general practitioner)

- Midwife

- Other:

**Lifestyle During Pregnancy**

**7. Are you physically active during pregnancy (at least 30 minutes, moderate to intensive activity)?**

- No, never

- Yes, occasionally

- Yes, 2-4 times per week

- Yes, (almost) daily

**8. How often do you drink coffee during pregnancy?**

- Daily, more than 3 cups

- Daily, between 1 and 3 cups

- Occasionally

- Never

**9. Do you currently smoke?**

- Yes

- No

**10. Did you smoke before your pregnancy?**

- Yes

- No

**11. Have you consumed alcohol since becoming aware of your pregnancy?**

- No, never

- Once per month or less

- 2-4 times per month

- Twice per week or more

**12. If you drink alcoholic beverages, how many drinks do you typically consume in a day?**

- 1-2

- 3-4

- 4-5

- 6 or more

**13. How often do you eat fatty sea fish (e.g., salmon, mackerel, herring)?**

- Daily

- Several times per week

- Once per week

- 1-2 times per month

- Never

**14. During your pregnancy, have you been mindful of eating what you consider to be a healthy diet?**

- Yes, very

- Yes, somewhat

- Hardly

- No

**15. Before pregnancy, how much attention did you pay to a healthy diet?**

- Very much

- Somewhat

- Hardly

- Not at all

**16. Have you tried to improve your diet compared to before your pregnancy?**

- Yes, very much

- Yes, a little

- Hardly

- Not at all

**17. If you have been pregnant before, how has your focus on nutrition changed this time?**

- I pay more attention to my diet than in previous pregnancies

- I pay less attention to my diet than in previous pregnancies

- No change

- This is my first pregnancy

**Nutritional Recommendations**

**18. Have you received advice on nutrition and/or dietary supplements during your pregnancy?**

- Yes, very detailed

- Yes, detailed

- Yes, a little

- No

**19. From whom did you receive this advice?**

- Gynecologist

- Other specialist (e.g., general practitioner)

- Midwife

- Nutritionist

- Alternative practitioner

- Other:

**20. Have you also informed yourself about this topic?**

- Yes

- No

**21. Please list the three most important sources from which you have obtained information on nutrition.** (Multiple answers possible)

- Books and brochures

- Prenatal classes (including online)

- Television

- Google search

- Internet forums

- Instagram

- Facebook

- TikTok

- YouTube

- Parents and family

- Friends and acquaintances

**22. Were you advised to fast or lose weight during pregnancy?**

- Yes

- No

**23. Who advised you on this?** (Multiple answers possible)

- Gynecologist

- General practitioner

- Midwife

- Parents, friends, or acquaintances

- Google search

- Internet forums

- Instagram

- Facebook

- YouTube

- Books, magazines, etc.

**24. Which of the following foods and beverages were you advised to avoid?** (Multiple answers possible)

- Pasta

- White flour products

- Raw fruits and vegetables

- Onions

- Garlic

- Ginger

- Mushrooms

- Eggs

- Milk and dairy products

- Animal products in general

- Raw or undercooked meat

- Raw milk and raw milk products

- Raw fish or seafood

- Game meat and offal

- Salami, tea sausage, minced sausage

- Parmesan cheese

- Antipasti

- Sweets

- (Refined) sugar

- Honey

- Chips and salty snacks

- Cinnamon, cardamom, cloves (including in holiday cookies or gingerbread)

- Poppy seeds

- Tonic water and bitter lemon

- Coffee

- Black tea

- Energy drinks

- Alcohol consumption

- Smoking/nicotine

- Other (please specify)

**25. Were you informed (or did you inform yourself) that coffee consumption during pregnancy should be limited to three cups per day?**

- Yes

- No

**26. Were you advised (or did you inform yourself) to avoid certain foods during pregnancy to prevent allergies in your child?**

- Yes

- No

**Supplement Intake**

The following section concerns the intake of dietary supplements. Please take the time to answer these questions carefully and refer to the packaging of your product if needed.

**27. Are you currently taking a vitamin or mineral supplement, or have you taken one at any point during your pregnancy?**

- Yes

- No

**Supplement Intake**

**28. Please select all the micronutrients you are or have been taking as supplements.**

**(Multiple answers possible)**

- Biotin (Vitamin B7)

- Calcium

- Choline

- Iron

- Folic acid

- Iodine

- Magnesium

- Omega-3 fatty acids (e.g., DHA, EPA)

- Vitamin A

- Vitamin B1

- Vitamin B2

- Vitamin B3

- Vitamin B5

- Vitamin B6

- Vitamin B12

- Vitamin C

- Vitamin D

- Vitamin E

- Vitamin K

- Zinc

**29. If you are taking an iron supplement, did you start it after being diagnosed with iron deficiency by a doctor?**

- Yes

- No

- I am not taking an iron supplement.

**30. For all nutrients listed below that you are taking or have taken, please indicate the daily dosage:**

- Folic acid (µg)

- Iodine (µg)

- Iron (mg)

- Omega-3 fatty acids (e.g., DHA, EPA) (mg)

**31. When did you start taking each of these nutrients? If you have already stopped taking them, please specify as well.**

- Folic acid

- Iodine

- Iron

- Omega-3 fatty acids (e.g., DHA, EPA)

**32. Who recommended these supplements to you?** (Multiple answers possible)

- Doctor

- Midwife

- Pharmacist

- Family, friends, or acquaintances

- Google search

- Internet forums

- Instagram

- Facebook

- TikTok

- YouTube

- Books, magazines, etc.

**33. Have you been diagnosed with a thyroid disorder (e.g., hyperthyroidism, hypothyroidism, or Hashimoto's thyroiditis)?**

- Yes

- No

**General Data**

**34. What year were you born?**

**35. What is your height (in meters) and weight (in kilograms) before your pregnancy?**

**36. What is your highest level of education?**

- No degree

- Secondary school certificate

- Intermediate school certificate

- High school diploma

- Completed vocational training

- Completed university degree

**37. What is the monthly net household income available to all household members after taxes and social security contributions?**

- Below €1,000

- €1,001–€1,500

- €1,501–€2,000

- €2,001–€2,500

- €2,501–€3,000

- €3,001–€3,500

- €3,501–€4,000

- Above €4,000

- I prefer not to disclose.

**Appendix 2**

**Description of the Study Population**

The detailed sociodemographic characteristics of the study population are presented in Table 1. Close to half of the respondents were pregnant for the first time (n = 1,604; 47.7%). It is noteworthy that the participants were highly educated, with 52.38% holding a university degree (108). However, the age, body mass index (BMI), and net household income of the respondents reflected the average pregnant population in Germany.

**TABLE 1.** Demographic and Socioeconomic Characteristics of the Study Population

| **Characteristic** | **Study Sample (n = 3,679)** |
| --- | --- |
| **Number of pregnancies** | **n (%)** |
| First pregnancy | 1,604 (47.7%) |
| Previous pregnancies | 1,759 (52.3%) |
| **Age** | **Years** |
| Mean (± SD) | 31.84 (± 3.9) |
| Median | 32 |
| Variance | 15.26 |
| Minimum–Maximum | 17–44 |
| **Body Mass Index (BMI)** | **(kg/m²)** |
| Mean (± SD) | 24.56 (± 5.21) |
| Median | 23.38 |
| Minimum–Maximum | 14.3–59.1 |
| **Education level** | **n (%)** |
| No formal degree | 1 (0.03%) |
| Secondary school certificate | 21 (0.62%) |
| Intermediate school certificate | 216 (6.42%) |
| High school diploma | 601 (17.87%) |
| Completed vocational training | 796 (23.67%) |
| University degree | 1,728 (52.38%) |
| **Monthly household net income** | **n (%)** |
| Under €1,000 | 38 (1.13%) |
| €1,001–€1,500 | 98 (2.91%) |
| €1,501–€2,000 | 202 (6.01%) |
| €2,001–€2,500 | 228 (6.78%) |
| €2,501–€3,000 | 300 (8.92%) |
| €3,001–€3,500 | 352 (10.47%) |
| €3,501–€4,000 | 500 (14.87%) |
| Over €4,000 | 1,329 (39.52%) |
| No response | 316 (9.40%) |

**Age Distribution**

Of the total 3,363 respondents, 3,358 provided valid information about their age. The resulting age distribution ranged from 17 to 44 years, with an average age of 31.84 years (SD ± 3.9). This aligns with the actual age distribution of all pregnant women in Germany (31.8 years; (109)), supporting the validity of the sample.

**BMI**

The BMI of 3,352 participants was calculated based on their height and weight. Eleven participants did not provide this information or provided invalid data. The average BMI was 24.56 (SD ± 5.21), and the median was 23.38. This parameter was collected to ensure the sample represents the population evenly. According to the Federal Statistical Office (108), the average BMI for the age group 20–40 years is 24.5.

More than 34.02% (n = 1,144) of participants were classified as overweight before pregnancy based on their BMI, and 12.10% (n = 407) were classified as obese. These values align well with the general population of German women, where 33.50% of women in the same age group are overweight, and 12.30% are obese (110).

**Educational Level**

To verify the validity of the sample regarding educational level, the highest educational attainment of the participants was surveyed. The distribution (Table 1) shows an above-average level of education compared to the German average for individuals under 45 years (108). However, notable differences exist within this age group: younger participants may still be in education, while older individuals, on average, have fewer tertiary degrees. Due to the uneven age distribution, exact congruence was not expected.

**Monthly Net Household Income**

The question regarding monthly net household income was answered by 3,047 respondents, while 316 did not provide this information. The questionnaire offered eight income intervals. For analysis, the income was grouped into two categories: below €2,500 net and above €2,500 net.

This categorization was chosen because, according to the OECD equivalence scale, the poverty threshold for a family with small children is €2,410 (53). The poverty threshold is defined as 60% of the median equivalized net income of private households.

**Appendix 3**

**Evidence-based avoidances**

Individuals with academic qualifications refrained from an average of 7.03 (SD ± 1.50) foods, whereas those without academic backgrounds refrained from 6.93 (SD ± 1.56) foods (p = 0.06). Income levels did not exhibit a significant correlation either, with those earning below €2,500 net income/month having an average of 6.84 (SD ± 1.68) and those earning above €2,500 averaging 7.01 (SD ± 1.50; p = 0.08). ). Income levels did not exhibit a significant correlation either, with those earning below €2,500 net income/month having an average of 6.84 (SD ± 1.68) and those earning above €2,500 averaging 7.01 (SD ± 1.50; p = 0.08).

Furthermore, women who placed a higher emphasis on nutrition during their pregnancy had a higher average of evidence-based avoided foods (7.03 [SD ± 1.53] vs 6.75 [SD ± 1.50], p < 0.001). A parallel trend was noted among women who received comprehensive dietary counseling from HCPs (7.25 [SD ± 1.31] vs 6.93 [SD ± 1.57], p < 0.001). Participants cared for by a midwife were more accurately informed about potentially hazardous foods than participants without midwife care, with an average of 7.42 (SD ± 1.02) compared with 4.95 (SD ± 1.83; p < 0.001, Figure 3). Moreover, women who informed themselves about nutrition topics indicated better adherence to the avoidance of the mentioned foods, averaging 7.08 (SD ± 1.49) compared with 6.54 (SD ± 1.65; p < 0.001) for women who did not inform themselves. Figure 3 presents the number of evidence-based avoided foods in correlation to midwife care.

**Non-evidence-based avoidances**

Similar to the previous category, no significant variances were observed based on education (academics: 1.93 [SD ± 2.15], nonacademics: 1.83 [SD ± 2.05]; p = 0.17) or income levels (income below €2,500 per month: 1.87 [SD ± 2.06], above €2,500: 1.89 [SD ± 2.11]; p = 0.87). Yet, women who prioritized a health-conscious diet during pregnancy tended to have higher values for non-evidence-based avoided foods (1.96 [SD ± 2.13] vs. 1.56 [SD ± 1.86]; p < 0.001). This trend was also evident among those who received comprehensive nutritional counseling (2.07 [SD ± 2.14] vs 1.82 [SD ± 2.08]; p = 0.01). Receiving care from a midwife was also linked to a higher likelihood of incorrect food avoidance, with an average of 1.99 (SD ± 2.1) compared with 1.36 (SD ± 1.8) without midwife care, which was a significant difference (p < 0.001). Similarly, self-informed individuals on nutritional topics also exhibited significant differences (2.04 [SD ± 2.1] vs. 1.23 [SD ± 1.68]; p < 0.001).
